# Recent advances in the discovery of small-molecule inhibitors of HIV-1 integrase

**DOI:** 10.4155/fsoa-2018-0060

**Published:** 2018-09-06

**Authors:** Eungi Choi, Jayapal Reddy Mallareddy, Dai Lu, Srikanth Kolluru

**Affiliations:** 1 Department of Biopharmaceutical Sciences, Keck Graduate Institute School of Pharmacy, 535, Watson Dr, Claremont, CA 91711, USA; 2 Department of Pharmaceutical Sciences, Texas A&M University School of Pharmacy, 1010 W. Avenue B, Kingsville, TX 78363, USA

**Keywords:** 3′-processing, AIDS/HIV, dual inhibitors, HIV integrase, HIV integrase Inhibitors, LEDGINs, strand transfer inhibitors

## Abstract

AIDS caused by the infection of HIV is a prevalent problem today. Rapid development of drug resistance to existing drug classes has called for the discovery of new targets. Within the three major enzymes (i.e., HIV-1 protease, HIV-1 reverse transcriptase and HIV-1 integrase [IN]) of the viral replication cycle, HIV-1 IN has been of particular interest due to the absence of human cellular homolog. HIV-1 IN catalyzes the integration of viral genetic material with the host genome, a key step in the viral replication process. Several novel classes of HIV IN inhibitors have been explored by targeting different sites on the enzyme. This review strives to provide readers with updates on the recent developments of HIV-1 IN inhibitors.

## Lay abstract

AIDS is an epidemic disease that endangers the lives of millions of people across the world. The AIDS virus, also known as HIV, has developed resistance to the majority of available drugs on the market, thus requiring the need for new drugs. HIV integrase is one of the key viral enzymes required for viral cell proliferation. Since there is no similar enzyme in the human body, major emphasis is being made to develop therapeutics for this novel target. The drugs that are at various stages of development for this target are reviewed here.

AIDS is an epidemic disease that endangers the health of millions of people worldwide. It is caused by the infection of pathogenic HIV. By the end of 2016, approximately 37 million people were living with HIV worldwide [1]. It was estimated that 1.0 million people died from AIDS-related complications in 2016 [1]. According to the HIV Surveillance Report from US Center for Disease Control (CDC), about 1.2 million Americans aged 13 years and older are suffering from HIV infection currently; of which 160,000 are unaware of their infection [2]. Since the clinical and biomedical characterization of AIDS in early 1980s, tremendous efforts have been made to identify effective therapeutics for this deadly disease. The first anti-HIV/AIDS chemotherapeutic agent azidothymidine was introduced in 1987, which reduces the progress of AIDS by inhibiting the enzyme reverse transcriptase (RT) that HIV uses to synthesize viral complimentary DNA. Since the identification of azidothymidine, several newer classes of anti-HIV/AIDS chemotherapeutic agents have been developed [3]. These agents are capable of arresting the HIV viral replication cycle at different stages including viral entry, fusion, reverse transcription, integration and protein maturation. With the marketing of these anti-HIV drugs, particularly the development of combinatorial antiretroviral therapy, the life span and quality of patients living with HIV improved significantly [4–6]. At present, anti-HIV chemotherapeutics have been pursued through various strategies by targeting different virus and host cell targets: inhibiting viral entry into cells by targeting the cell CD4 receptor; inhibiting virus adsorption to the host cells by targeting viral envelope glycoprotein gp120; inhibiting viral entry by targeting cellular CXCR4 or CCR5 co-receptors; inhibiting virus–cell fusion by targeting viral glycoprotein gp41; inhibiting HIV RT by targeting its substrate-binding site and non-substrate-binding (allosteric) site; inhibiting the integration of the proviral DNA into the host cell genome by targeting HIV integrase (IN); inhibiting proviral DNA transcription and transactivation by targeting cellular transcriptional factors; and inhibiting virus maturation and infectivity by targeting HIV protease (PR) [3,7]. Although about 31 anti-HIV drugs have been developed, several issues have challenged clinical management of HIV infection. These include the emergence of multidrug-resistant strains, harsh adverse effects of the antiviral drugs, requiring high degree of patient's compliance and significant expenses of the treatment [8]. Collectively, these issues press the need to pursue novel anti-HIV therapeutics (Figure 1).

**Figure 1.**
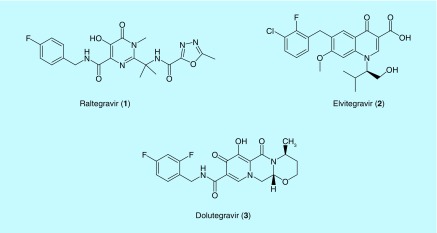
**Clinically used HIV integrase inhibitors.**

HIV requires three essential enzymes for its replication, namely, RT, PR and IN. By far, most of the anti-HIV drugs on the market are inhibitors of HIV RT and PR enzymes. HIV IN is a relatively newer therapeutic target. Its therapeutic value was proven with the discovery of raltegravir (RAL), the first IN inhibitor (INI) for human use [9]. In comparison to other viral targets, the HIV IN stands out as a unique antiviral target because it has the least resemblance to any human protein. This unique feature makes IN an attractive anti-HIV target for reducing off-target side effects. More recently, two other HIV INIs, elvitegravir (EVG) [10] and dolutegravir (DTG) [11], were approved by the US FDA for the treatment of AIDS. RAL is a potent and well-tolerated anti-HIV drug. However, it has the twice-daily dosing limitation and a relatively low barrier to the development of resistance. EVG combined with other antiviral agents achieved the benefit of once-daily dosing, but it suffers from extensive cross-resistance with RAL [12]. DTG is the newest INI approved by the FDA. It offers good tolerability, once-daily dosing without a pharmacological enhancer and relatively low cross-resistance with RAL. It is superior to earlier generation INIs when used in antiretroviral-experienced patients [13]. The development of novel INIs will extend the treatment options for patients with HIV-1 infection. Thus, tremendous research efforts have been made in the field to identify novel chemical entities for the class of HIV INIs. This review provides a synopsis of HIV IN and an overview of new advances in the discovery of small-molecule HIV INIs.

## Mechanism of viral integration

**Figure 2.**
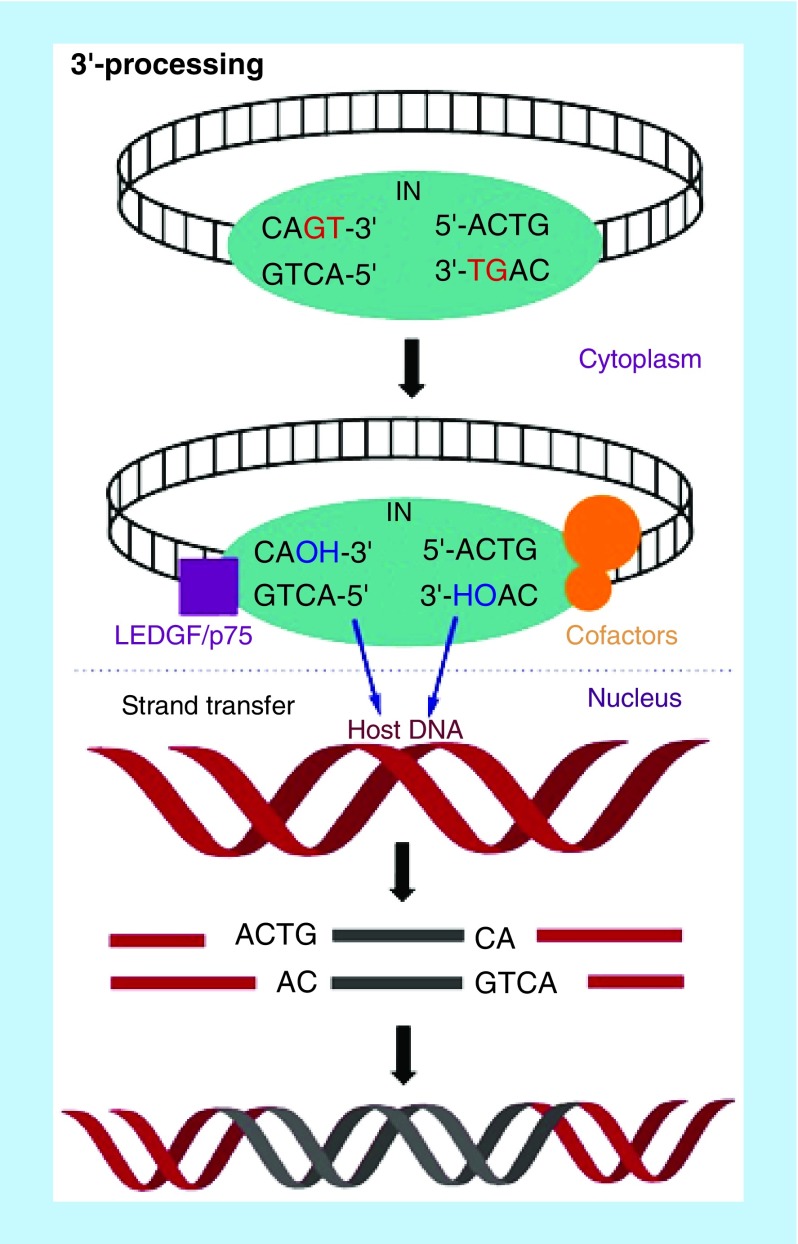
**Mechanism of HIV integration.** **3′-Processing:** Integrase locates CAGT tetranucleotide at both 3′-ends of viral cDNA and trims off to expose sticky CA dinucleotide; **Strand transfer:** The trimmed viral DNA-IN complex combines with cofactors to form PIC, which translocates into the nucleus and integrates with host DNA. IN: Integrase; PIC: Preintegration complex.

Integration of HIV genetic materials with the host cell genome is essential for viral replication. Upon entry into the host cells, the viral RNA is reverse transcribed into double-stranded cDNA by the viral RT enzyme. Following reverse transcription, the viral cDNA is primed for integration with the host DNA. The integration process is carried out by the viral IN enzyme by forming a complex with viral cDNA in cytoplasm along with cellular cofactors to complete the integration process in the host nucleus. The viral genomic integration takes place in two consecutive steps: first the viral cDNA 3′-processing (3′-P) in the cytoplasm followed by strand transfer (ST) in the host cell nucleus [14,15]. In the first step, IN locates CAGT tetranucleotide sequence in the long terminal repeats (LTRs) of the 3′-end of viral cDNA and trims off two nucleotides (i.e., GT dinucleotides) to expose CA-3′-OH at both 3′-ends of viral cDNA. The trimmed viral cDNA remains bound to the IN and forms a pre-integration complex (PIC) with viral and host cofactors, which is then translocated to the nucleus to integrate with the host cell DNA. During ST step, the exposed CA-3′-OH of viral DNA acts as a nucleophile to attack the phosphodiester bonds on complementary strands of the host DNA through a transesterification reaction. This reaction is facilitated by two divalent metal ions, which help to stabilize the IN-DNA complex [16]. In this process, ST takes place concomitantly for both extremities of viral DNA. The 5′-ends of the viral DNA and the 3′-ends of host DNA at the insertion points remain uncoupled in the integration intermediate [17]. This leaves a five-base, single-stranded gap at each junction between the inserted viral DNA and the host DNA, and an unpaired two-base fragment at the 5′-ends of the viral DNA. Completion of integration requires cellular enzymes to remove the unpaired nucleotides at the 5′-ends of the viral DNA and repair the single-strand gaps between viral and host DNA [18] (Figure 2).

## Structure of HIV IN

HIV IN is a 32 kDa protein encoded at the 3′-end of the *HIV Pol* gene [19]. This multifunctional enzyme contains three structurally and functionally distinct domains: a zinc-binding N-terminal domain (NTD; residues 1–50), a catalytic core domain (CCD; residues 51–212) and a nonspecific DNA-binding domain (CTD; residues 213–288) [18]. The NTD contains highly conserved His and Cys residues (the HHCC motif), which chelate one zinc ion. The CCD contains the endonuclease and polynucleotidyl transferase sites with its three highly conserved acidic residues: Asp64, Asp116 and Glu152. These three residues can bind one or two divalent metal ions, such as Mn^2+^ or Mg^2+^. The DDE (Asp-Asp-Glu) motif is conserved among all retroviral IN proteins and is instrumental for the catalytic activity of IN. Mutation of any of these residues abolishes or diminishes the catalytic activity. The CCD consists of five β-sheets and six α-helices, and folds into a dimer with twofold symmetry. The CTD domain binds DNA substrates non-specifically and is responsible for stabilizing the complex once bonded with DNA. Although the roles of the NTD and the CTD are less well understood in comparison with CCD, all three domains are essential for catalysis of 3′-P and DNA ST [20,21]. The atomic structures of NTD [22], CCD [23] and CTD [24] were determined either by solution NMR or by x-ray crystallography. Due to the insolubility of the full-length HIV IN protein, its crystal structure remains unknown. Recently, a crystal structure of full-length IN from the prototype foamy virus in complex with its substrate DNA was resolved [25]. All of these structural information indicated that HIV IN most likely exists as a dimer or in higher oligomeric states, such as a tetramer in solution [26].

Integration of viral DNA with host DNA is a complex process that depends on the import of PIC into host cell nucleus. PIC not only contains viral proteins that include matrix protein p17, capsid protein 24, the nucleocapsid protein p7/p9, the viral RT, IN and accessory protein Vpr [21] but also comprises a number of cellular proteins such as barrier-to-autointegration factor (BAF), IN interactor 1 (INI1), p300 histone acetyl transferase (HAT), Lens Epithelium-Derived Growth factor (LEDGF/p75) and high-mobility group protein A1 HMG-I(Y), among others [18,21,27]. The viral proteins present in PIC facilitate its transport into host cell nucleus [18,28]. On the other hand, cellular proteins packaged within PIC might directly influence catalytic activities of IN as well as impact non-catalytic aspects of PIC such as stability, nuclear import and binding to specific regions of chromatin [27].

## HIV INIs

HIV INIs added new armament to antiretroviral therapy since they target a distinct process (i.e., integration) in the retroviral life cycle. Because of their unique mechanism of action, they have potential to reduce the chance of adaptation by the virus either when used alone or used in combination with other types of anti-HIV drugs. The structural complexity of IN and its mechanism of action suggested several strategies for therapeutic interventions: interfering the binding of IN to viral cDNA ends; interfering IN oligomerization; interfering the 3′-P activity; interfering the ST activity; and interfering with the protein–protein interactions between IN and cellular cofactors [15,29,30]. In the journey of discovery of therapeutically useful INIs, natural products have provided numerous leads that include caffeic acid phenethyl ester, chicoric acids and funalenone, among others [31,32]. Notably, many of these natural products showed inhibitory effects on other antiviral targets such as RT, PR and gp120 and structurally characterized with metal-chelating hydroxyl groups [31]. Searching IN-specific inhibitors had been focused on identification of compounds that target the IN active site, particularly with the mechanism of action as metal chelators. Such IN-specific inhibitors generally can be classified into two groups: inhibitors that can suppress the 3′-P and ST (often referred to as 3′-P inhibitors), and inhibitors selective for the IN ST activity (INSTIs). The 3′-P inhibitors have the preference to prevent viral DNA binding to IN. In contrast, INSTIs bind to the IN-viral DNA complex and probably interfere with the binding of host cellular DNA [29,31]. The 3′-P inhibitors are represented by styrylquinolines [33] and antimicrobial peptide indolicidin [34]. As discussed in the subsequent sections, INSTI discovery was the most successful when compared with 3′-P inhibitors. However, they also developed resistance very rapidly due to viral mutations. It is necessary to revisit the development of 3′-P inhibitors to address these resistance problems effectively. Recently, a study describing the importance of Q146 amino acid interaction with viral DNA for 3′-P is reported, providing newer insights to develop 3′-P inhibitors [35]. Several classes of INIs that are effective against both ST and 3′-P are reported [36,37]. Optimizing 3′-P inhibitors by improving their interaction with Q146 may provide novel leads. This review will focus on small-molecule INSTIs that lead to clinical candidates.

ST inhibition is the most productive approach by far that leads to clinical candidates. Three INSTIs have been approved by the FDA as new anti-HIV drugs. As is successful for many drug discovery programs, massive screening of compound libraries identified the first type of lead compounds for INIs. The identification of 5CITEP (**4**) by Shionogi [38,39] and L-708,906 (**5**) by Merck [40,41] provided the prototypical scaffold (β-diketo acid [DKA]) for many INIs developed later. The DKAs offered innovative tools for the investigations of the drug-target interaction, which pave the way for the discovery of first-generation INIs for clinical application.

## Early INIs from DKA & its derivatives

The discovery of DKA scaffold as INIs was initiated by the identification of 1-(5-chloroindol-3-yl)-3-hydroxy-3-(2*H*-tetrazol-5-yl)propenone (5-CITEP) (**4**) [38]. This compound featured an α,γ-diketo acid functionality, of which the acid was replaced with a bio-isosteric tetrazole. 5-CITEP (**4**) was the first INI that has been co-crystalized with the IN CCD domain [38]. The inhibitor binds centrally in the active site between the two aspartates, D64 and D116, and the glutamate E152, and makes a number of close contacts with the protein to compete with viral DNA for binding to IN. 5-CITEP (**4**) inhibited IN 3′-P and ST at IC_50_ values of 35 and 0.65 μM, respectively [42].

Soon following the discovery of 5-CITEP, Merck disclosed L-708,906 (**5**) [40] and L-731,988 (**6**), of which L-731,988 exhibited potent activity against HIV-1 IN *in vitro* and had a remarkable selectivity for ST (IC_50_ = 80 nM for ST and IC_50_ = 6 μM for 3′-P) [42]. Compounds **5** and **6** are potent against variants that are resistant to HIV RT and PR inhibitors. However, when the compounds were tested against persistently infected cells, they failed in suppressing virus production at the concentration up to 50 μM [40]. Following the initial work on L-708,906 and L-731,988, Merck discovered a series of derivatives of 8-hydroxy-[1,6]-naphthyridine-7-carboxamides that represented by L-870,810 (**7**) and L-870,812 (**8**) [43], of which inhibitor **7** exhibited potent inhibition against IN ST activity (IC_50_ = 8 nM) and robust antiviral activity with an EC_95_ value of 15 nM in a cell-based assay [44]. Both compounds were the first INSTIs that showed anti-HIV activity in experimental animal models. L-870,810 (**7**) had improved bioavailability (i.e., oral bioavailability >60% and half-life ∼5 h in rhesus macaques) compared with earlier DKA-based INSTIs, which made it possible to enter clinical trials. However, the liver and kidney toxicity that appeared after long-term use in dogs halted the drug candidate for further clinical development [31,45]. Another early INSTI that entered clinical trials was the DKA derivative (*Z*)-1-[5-(4-fluorobenzyl)furan-2-yl]-3-hydroxy-3-(1*H*-1,2,4-triazol-3-yl)propenone (S-1360) (**9**), in which a triazole moiety is the bioisostere of the carboxylate group in DKA scaffold. Compound **9** exhibited a 20-nM IC_50_ for IN inhibition *in vitro*, and it also inhibited HIV replication in MTT assays with EC_50_ and CC_50_ values of 200 nM and 12 μM, respectively. The compound was developed by Shionogi (Osaka, Japan) and GlaxoSmithKline (London, UK), and was the first INI that went on clinical trials for the indication of HIV infection [46]. Preclinical studies of S-1360 (**9**) showed plausible pharmacological, pharmacokinetic, safety and toxicology results in animal models. However, this candidate failed to deliver efficacy in humans due to its rapid metabolism and clearance via a non-CYP450 clearance pathway including reduction and glucuronidation [47]. Later, Shionogi and GlaxoSmithKline discovered another INSTI GSK364735 (**10**) [48], which is 4-hydroxy-2-oxo-1,2-dihydro-1,5-naphthyridine. Compound **10** inhibited HIV replication with robust potency. Its EC_50_ values were 1.2 and 5 nM, respectively, in peripheral blood mononuclear cells (PBMCs) and MT-4 cells. This compound was equally active against wild-type viruses and viruses resistant to approved RT or PR inhibitors, whereas it showed some cross-resistance with other INIs [48]. However, clinical development of **10** was terminated early due to hepatotoxicity in long-term safety studies in monkeys, although no significant side effects were observed [49].

To understand the role of divalent metal chelating in the inhibition of IN dimeric diketo acid containing inhibitors were synthesized with amide or benzyloxy as the linker. It was concluded that two diketo subunits separated by uniquely designed linkers can potentially chelate two metal ions provided from one IN active site or two active sites in a higher order tetramer. Most of the compounds had nanomolar inhibitory effects on the ST step and were low micromolar inhibitors of the 3′-P step [50]. Later, some triketo acid inhibitors of HIV-1 IN were reported, but these molecules only had moderate activities that were weaker than those molecules containing a diketo acid [51].

Although the aforementioned INSTIs failed at different stages during drug development, they are instrumental for understanding the interactions between the HIV IN and its inhibitors targeting the IN active site. According to the different crystal structures of IN CCD, the integration process most likely involves two divalent magnesium ions, although the number of metals required for IN activity has not been fully characterized yet. It was postulated that the first metal cation is coordinated by the two catalytic residues D64 and D116, whereas the second metal cation is coordinated by D116 and E152 [52,53]. The metal ions are essential for the assembly of stable IN-DNA complex and as catalytic cofactors. DKA derivatives are characterized with structural elements of a γ-ketone, an enolizable α-ketone, and a carboxylic acid, of which the carboxylic acid can be substituted with either acidic (e.g., tetrazole and triazole) or basic (e.g., pyridine) bioisosteres. They have been found to compete with target DNA for IN catalytic site of IN-viral DNA complex. DKA derivatives exert their antiviral activities through chelating the metal ions in the active site [52–54]. Their general structural features include a coplanar arrangement of three heteroatoms that can chelate the two catalytically important metal ions at the IN active site, which are tethered by a conserved D64, D116 and E152 motif in HIV IN. The early INIs from the class of DKA derivatives lay a foundation for the discovery of three INSTIs approved by the FDA. In particular, their preclinical and clinical studies provided proof-of-concept for the feasibility of using INIs as antiretroviral therapy.

## INIs approved for clinical application

### First-generation INIs

#### Raltegravir

RAL (**1**) was the first in the class of INIs being approved by the FDA for the treatment of HIV infection. It was initially approved for use in antiretroviral treatment-experienced adult patients [55–58]. Although it was discovered by Merck from the evolution of DKAs in their HCV polymerase program, the compound is completely inactive on the HCV polymerase. RAL is a potent, reversible and selective INSTI showing nanomolar activity in the enzymatic ST assay (IC_50_ = 0.085 μM) [9]. It is administered orally at the dose of 400 mg twice-daily. In HIV-infected patients with limited treatment options, RAL plus optimized background therapy provided better viral suppression than optimized background therapy alone for at least 48 weeks [59]. In treatment-naive patients with plasma HIV-1 RNA levels greater or equal to 5000 copies/ml and CD4 T-cell counts greater or equal to 100 cells/mm, the drug leads to plasma HIV-1 RNA levels less than 400 copies/ml in 85–98% of patients and less than 50 copies/ml in 85–95% of patients by 24 weeks of treatment [60]. In addition to the high clinical efficacy, initial use of RAL also showed good tolerability, a favorable safety profile and absence of significant drug–drug interactions [55,58]. It is unique mechanism of action at time of approval made RAL a great addition for the treatment of patients with extensive drug resistance to other anti HIV drugs such as inhibitors of HIV RT and PR. Because of its relative lack of drug interactions, generally low adverse effect profile and novel mechanism of action, RAL is a landmark in the development of anti-HIV drugs. The proven clinical effectiveness of RAL confirmed that HIV IN is a viable target for anti-HIV chemotherapy. Although RAL soon became a blockbuster anti-HIV drug after it was approved in 2007, viral strains with multiple mutations in IN CCD have been identified that render the drug ineffective against these viruses. The mutations that were identified after RAL treatment include Q148/H/K/R, E138A/K, G140A, T66A, Q95K, Y143C/R, N155H [61]. Three signature resistant-associated mutations Y143C/R ± T97A, Q148H/K/R ± G140S/A and N155H ± E92Q have been identified. Acquisition of Q148H/K/R, Y143C/R or N155H reduced susceptibility to RAL for a more than tenfold decline [55], whereas acquisition of Q148K, E138 and G140A mutations reduced susceptibility to RAL by several hundred fold. Newer INIs need to develop inhibitors to address resistance to these known mutations. For example, pyrrolyl DKAs developed by Corona *et al*. are effective against RAL-resistant strains. The site-directed mutagenesis studies of these compounds showed that they interact with P145, Q146 and Q148 amino acids. They were shown to have activity against RAL-developed Y143A, and N155H-mutant strains [62,63]. Several newer classes of drugs addressing these mutations are discussed in the appropriate drug classes in the subsequent sections of this review.

#### Elvitegravir

EVG (**2**) is the second INI approved by the FDA. It was initially developed by the Central Pharmaceutical Research Institute of Japan Tobacco, Inc., (Osaka, Japan) and later licensed to Gilead Sciences (CA, USA) for clinical development. It was approved as a component of a fixed-dose preparation, which combines EVG, cobicistat (COBI), which is a pharmaco-enhancer, and two nucleoside/nucleotide RT inhibitors, emtricitabine (FTC) and tenofovir disoproxil fumarate (TDF) [12,64]. The multicomponent medication is commercialized as a single-tablet under the brand name STRIBILD^®^ (EVG 150 mg/COBI 150 mg/FTC 200 mg/TDF 300 mg [EVG/c/FTC/TDF]) which is the first INI-based single-tablet regimen administered once-daily. In the USA, it has been approved for use in treatment-naive HIV patients, but not for treatment-experienced patients. In Europe, STRIBILD was approved not only for treatment-naive HIV patients but also for use in patients who do not show resistant to any of the antiviral agents contained in the STRIBILD preparation. Later the FDA-approved Genvoya^®^ as a single-tablet regimen for HIV treatment with combination of EVG, COBI, FTC and nucleoside/nucleotide RT inhibitors tenofovir alafenamide (TAF). Genvoya was made up with combination of EVG 150 mg/COBI 150 mg/TAF 10 mg/FTC 200 mg (EVG/c/TAF/FTC) [12,64]. In the USA, it has been approved for use in treatment-naive HIV patients and virologically suppressed patients. The lower-dosed single-tablet regimen has significantly reduced bone and renal side effects compared with STRIBILD treatment [65]. EVG was derived from quinolone antibiotics, and used the 4-quinolone-3-carboxylic acid in lieu of the diketo acid motif. This scaffold retained three chelating groups, including the carbonyl of the quinolinone ring. It possesses potent inhibitory activity with an IC_50_ of 7.2 nM in the ST assay, and shows an EC_50_ of 0.9 nM in an acute HIV-1 infection assay [66,67]. Clinical trials showed that the Genvoya regime was very effective in reducing viral load (plasma HIV-1 RNA levels) to less than 40 copies/ml at the week 48 assessment, and displayed durable suppression of viral load to less than 50 copies/ml for up to 6 months [68]. The discovery of EVG and the development of Genvoya brought in an important advance with once-daily single-tablet regime for the effective treatment of HIV-1 infection. In comparison with multiple-tablet regimens and multiple dosing regimens, Genvoya can significantly improve patient's compliance and treatment adherence, which is one of the instrumental factors for the success of treating HIV-1 infection. However, EVG exhibits a moderate genetic barrier to IN resistance as RAL does [69,70]. The primary resistance mutations associated with the failure of RAL-based treatments often lead to cross-resistance to EVG. This abolished the possibility to use EVG-based therapies when RAL-based treatment fails. Additionally, the Genvoya regime requires the use of a pharmacokinetic enhancer such as COBI to inhibit CYP3A4, which is the primary enzyme to metabolize EVG.

### Second-generation INIs

#### Dolutegravir

The low generic barrier of HIV in developing mutations resistant to RAL and EVG presses the need of developing newer HIV INIs that have limited or no-cross resistance to early generation INIs (**1** and **2**) or other classes of antiviral drugs. DTG (**3**), the latest INI approved by the FDA answered this call. This antiviral INSTI was discovered and developed by Shionogi and GlaxoSmithKline [71,72] and marketed by GSK as a 50 mg tablet under the brand name Tivicay^®^. It was approved for use in HIV-infected adults who are treatment-naive and treatment-experienced, including those who have been treated with other INSTIs. DTG (**3**) is also approved for use in children ages 12 years and older weighing at least 40 kg who are either treatment-naive or treatment-experienced, except those who have previously treated with other INSTIs [11,73]. In a variety of cellular antiviral assays, DTG robustly inhibited HIV replication at low nanomolar or subnanomolar potency. For instances, its EC_50_ against HIV-1 was 0.51 nM in PBMCs, 0.71 nM in MT-4 cells and 2.2 nM in a PHIV assay. Its mechanism of action as an INSTI was further demonstrated by a variety of *in vitro* assays including its potent inhibition of INST activity with an IC_50_ of 2.7 nM [72]. DTG is obtained from the optimization of a series of carbamoyl pyridone analogs, which were designed through a two-metal chelation model of the IN catalytic active site [71]. The tricyclic carbamoyl pyridine motif of DTG provides its oxygen-derived lone pairs as hard base to form optimal chelation with the two divalent metal ions in the IN active site [71]. The carbonyl of the C-5 carboxamide on DTG is not involved in the metal chelation, whereas it renders DTG more structural flexibility that allows DTG to be more embedded into the hydrophobic pocket of the IN active site when compared with other INSTI. Additionally, DTG could readjust its position and conformation in response to structural changes in the mutant IN active site that became resistant to RAL [74]. A therapeutic advantage of DTG is its ability to maintain high potencies against mutant strains of HIV that are resistant to RAL and EVG [11,73].

#### GS-9160

**Figure 3.**
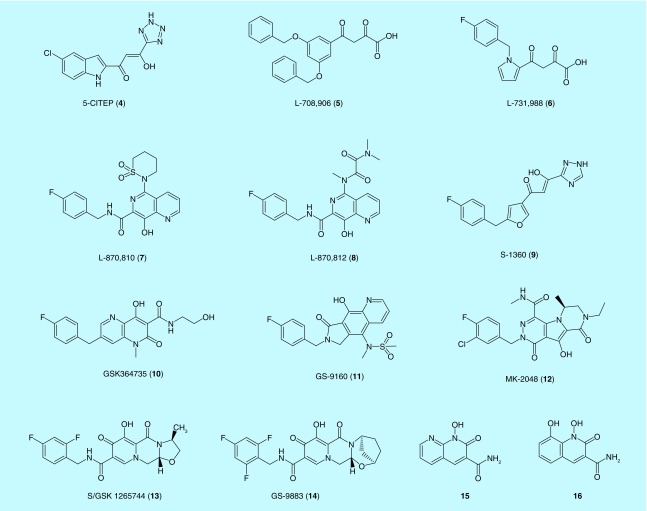
**Representative structures of HIV integrase inhibitors binding at its active site.**

GS-9160 (**11**) Figure 3 is a tricyclic INI containing 8-hydroxyquinoline moiety, as reported by Gilead Sciences [75]. It was discovered to mimic the active conformation of **7** by including tricyclic pharmacophore, in order to increase the binding affinity [76]. The compound is also a structural analog of a 4-quinolone-3-carboxylic acid-based inhibitor, EVG [77]. GS-9160 (**11**) showed a potent and selective antiviral activity at nanomolar range, but its potency decreased from six- to tenfold in the presence of human serum [76]. Due to its poor pharmacokinetic profile and unfavorable bioavailability, development was halted after Phase I clinical trials [75–77]. Viral resistance to **11** was observed with mutations at E92V and L74M in the CCD of IN. These mutations were also seen with L-870 810 (**5**), RAL (**1**) and EVG (**2**) suggesting that these compounds interact in a similar fashion at the IN active site [76].

#### MK-2048

MK-2048 (**12**) was developed by Merck & Co. by optimizing a tricyclic 10-hydroxy-7,8-dihydropyrazinopyrrolopyrazine-1,9-dione compound, which selectively inhibits ST step at nanomolar IC_95_ values and favorable pharmacokinetics in animal models [75]. It has a tricyclic hydroxypyrrole that contains a metal-binding pharmacophore and a halogenated benzyl moiety [78]. It exhibited an inhibitory activity four-times longer than RAL, and has shown to be potent against first-generation drug-resistant viruses with mutations like N155S and G140S/Q148H [79–81]. Selection studies in cultures revealed mutations at G118R and E138K conferred an eightfold resistance to MK-2048 [75], although it did not confer resistance to either RAL or EVG [79]. As a result, it was proven that second-generation INSTI resistances follow a different pathway to their first generation counterparts [79]. Currently, MK-2048 (**12**) is undergoing Phase II clinical trials [82].

#### Cabotegravir

Cabotegravir (**13**) also known as S/GSK 1265744, or GSK-744, was developed by Shionogi-ViiV Healthcare and GSK [83]. It is a part of the carbamoyl pyridone class of compounds and is a structural analog of DTG (**3**) [84,85]. In addition to its nanomolar IC_50_ values *in vitro*, cabotegravir (**13**) has a high genetic barrier to resistance *in vitro* as well as a good pharmacokinetic profile with a long half-life of 30 h that allows a low-dose, once-daily oral dosing or a monthly to quarterly parenteral dosing using a nanosuspension formulation [86]. Cabotegravir does not require coadministration with a CYP450 (CYP3A) isozyme inhibitor like DTG [85], and was shown to be well-tolerated at all dose levels with no clinical evidence of viral resistance [87,88]. It shares a similar drug resistance profile with DTG (**3**), but retains activity against RAL (**1**) and EVG (**2**) resistant mutants [89]. Currently, cabotegravir is undergoing Phase III clinical trials.

#### Bictegravir

Bictegravir (BIC), also known as GS-9883 (**14**), was a potent inhibitor of IN with low nanomolar IC_50_ (7.5 nM) [90,91]. BIC has displayed improved resistance compared with RAL, EVG and DTG, particularly for high-level INSTI resistance containing combinations of mutations such as E92Q+N155H or G140C/S+Q148R/H/K. In addition to high resistance barrier, BIC exhibited synergistic *in vitro* antiviral effects in combinations with the nucleos(t)ide RT inhibitors TAF, FTC or the PR inhibitor darunavir [90]. Later, Gilead Sciences developed a single-tablet anti-HIV medication (Biktarvy^®^) combining BIC with FTC and TAF [92]. The Biktarvy was approved by the FDA for use in the USA and globally in February 2018 for patients who did not previously receive antiretroviral treatment or to replace the current antiretroviral regimen in those who have achieved virological suppression (HIV-1 RNA/50 copies/ml) for ≥ 3 months.

#### Bicyclic 1-hydroxy-2-oxo-1,2-dihydropyridine-3-carboxamide-containing INIs

Second-generation DTG (**3**) has a halobenzyl group appended via an amide carbonyl proximal to but not part of the metal-chelating heteroatom triad, like the halobenzyl amide carbonyl of other INSTIs. It is hypothesized that the halobenzyl linker moiety is more flexible, and allows DTG (**3**) to bind tightly to wild-type and mutant IN-DNA complexes. However, both DTG and other INSTIs have a hydroxyl group as the central component of the triad. Novel inhibitors employ a hydroxyl amide group that serves as the central high-affinity metal-chelating component of the triad. The amide carbonyls of halobenzyl amide moieties therefore were found to be not required in metal chelation [93].

On this basis, a series of bicyclic inhibitors were developed that include 1-hydroxy-2-oxo-1,2-dihydro-1,8-naphthyridine-3-carboxamide (**15**) and 1,8-dihydroxy-2-oxo-1,2-dihydroquinoline-3-carboxamide (**16**) ring systems. Halobenzylamide functionality is appended through a linker whose carboxamide carbonyl is not an obligatory component of the key metal-chelating heteroatom triad. The series exhibited single-digit nanomolar EC_50_ values against wild-type IN in cell-based assays. In addition, some of the compounds had greater antiviral efficacies compared with RAL (**1**) against a panel of IN mutants with single mutations Y143R, N155H, G118R and the double mutations G140S/Q148H and E138K/Q148K [93].

### Allosteric INIs

The clinically approved INSTIs function by targeting the IN active site. Many of these candidates are suffering from resistance and cross resistance issues since they all share same mechanism of action. Recent developments to overcome resistance problems by targeting novel sites or functioning through novel mechanisms have resulted in the design of allosteric INIs (ALLINIs). ALLINIs bind to a site topologically distinct from the catalytic site and inhibit protein–protein interactions of HIV IN and its essential cellular cofactors [94]. When ALLINIs bind to an allosteric site, they induce a major conformational change to the overall structure of the catalytic site [95]. Since ALLINIs target a site distinct from the active site, they have a resistance profile generally different and non-overlapping with INSTIs [96].

Several ALLINIs bind at the IN CCD interface and function by dual mechanisms. Many ALLINIs have shown to tether the subunits of IN together, stabilizing the enzyme and decreasing the dynamic flexibility between IN subunits, a property of IN that is required for its function. Thus, in this way, ALLINIs cause IN to become catalytically inactive. This mechanism of inhibition has been termed ‘aberrant IN multimerization’, which means these compounds cause the assembly of IN into a unit that is catalytically inactive as it no longer can bind viral DNA and integrate it into host DNA [97].

#### LEDGF-IN interaction inhibitors

The majority of ALLINIs target the LEDGF/p75, which was discovered as the first cellular cofactor crucial for the function of IN to merge viral cDNA with host genome. LEDGF/p75 works to enhance the interaction between PIC and the host chromosome [98]. x-ray crystal structure and site-directed mutation studies discovered that LEDGF/p75 binds to IN at the integrase binding domain (IBD) in its C-terminus through the residues, Ile365, Asp366, Phe406 and Val 408. It also binds to the chromatin through N-terminal PWWP (Pro-Trp-Trp-Pro), which helps localize the site of interaction, with the help of A/T-hook elements [99,100].

Using the fact that LEDGF IBD binds to a defined pocket at the interface of two IN CCD dimers, several small molecules have been developed that target IN-LEDGF/p75 interaction. These novel ALLINIs were consequently called LEDGINs [101]. LEDGINs bind to the IN dimer and stabilize it, affecting oligomeric flexibility and intasome formation [100]. By modulating multimerization required for enzymatic activity, LEDGF/p75, and thus LEDGINs, have the ability to allosterically affect IN activity [15,102,103]. In addition, LEDGINs also induce a decrease of deletions at the 2-LTR junctions in the 2-LTR circles produced after HIV-replication, which is consistent with the 3′-P inhibition step of INSTIs [103]. By inhibiting both LEDGF/p75 interactions and catalytic activity, LEDGINs can inhibit both ST and 3′-P at the same level unlike INSTIs [104].

Because the LEDGF/p75-binding pocket at the CCD dimer interface is distant from the active site, a new way to overcome the cross-resistance problem associated with active-site targeting INSTIs has opened [105]. LEDGINs are not cross-resistant with INSTIs. The resistance strength varies depending on LEDGINs potency and initial resistance selection has found resistance to LEDGINs with a single mutation to multiple mutations [103].

It was also discovered that LEDGINs and INSTIs act in an additive or synergistic way, opening up possibilities of LEDGIN/INSTI combination therapy. In addition, virological characterization of cyclic peptides bonded to LEDGF/p75 in cell culture showed that LEDGINs not only block integration but also impair the infectivity of viral particles [103].

#### D77

The first small-molecule LEDGIN was discovered through high-throughput screening is 4-[(5-bromo-4-{[2,4-dioxo-3-(2-oxo-2-phenylethyl)-1,3-thiazolidin-5-ylidene]methyl}-2-ethoxyphenoxy)methyl]benzoic acid (D77) (**17**), which belongs to the benzoic acid derivatives [75]. It binds to the interface between IN CCD and LEDGF IBD by occupying hydrophobic pocket of IN-inhibiting HIV-1 IN interaction with LEDGF/p75. Compound **17** has IC_50_ value in the micromolar range for LEDGF/IN interaction but it exhibits cytotoxicity [106,107]. It was found unable to affect HIV-IN dimerization like other potent LEDGINs. It was implied to follow a different mechanism of action. In addition, **17** failed to inhibit LEDGF/p75-IN interactions in AlphaScreen assays but worked in yeast two-hybrid-based cellular assays. In a site-directed mutagenesis using SPR assays, mutations T125A, Q95A, W131A and T174A reduced the ability of **17** to bind to IN CCD [107]. Another compound discovered to inhibit IN-LEDGF interaction is 1,4-bis(5-(naphthalen-1-yl)thiophen-2-yl)naphthalene (**18**) [99,105,106,108].

#### BI-1001

The approach of structure-based drug design led to the discovery of a series of 2-(quinolin-3-yl)acetic acid derivatives that inhibited IN-LEDGF/p75 binding and HIV-1 replication in cell culture [109]. These molecules are composed of four key pharmacophoric features: a quinolone ring, a phenyl ring, a carboxylic acid and an methoxy group at the α-position of the carboxylic acid [110].

This class of compounds block the formation of stable synaptic complexes between IN and viral cDNA by allosterically stabilizing the inactive multimeric form of IN. In addition, they inhibit LEDGF binding to the stable synaptic complex. This multimodal mechanism results in cooperative inhibition of integration of viral cDNA and HIV-1 replication in cell culture [111]. The most potent compound of the series is BI-1001 (**19**) that exhibits a micromolar IC_50_ value and is currently being developed by Gilead Sciences Inc. [110]. Feng *et al*. performed resistance studies using the mutant A128T and found that the threonine substitution conferred an increase in compound **19** inhibition for ST, 3′-P and LEDGF/p75 interaction [112].

#### 3-Hydroxypicolinamides

Structure–activity relationship studies on the 2,3-dihydroxybenzamide scaffold led to the identification of the selective IN-LEDGF/p75 inhibitor, LZ-14 (**20**) [113]. Further optimization of **20** by merging the oxidation-liable aniline amino group into the phenyl ring resulted in 3-hydroxypicolinamide-based (**21**), a novel LEDGIN [114]. 3-Hydroxypicolinamide derivatives disrupt IN-LEDGF/p75 interactions by forming several H-bonds and hydrophobic interactions with important allosteric site residues, such as Glu170, His171, Thr174, Trp132, Ala128. These derivatives block HIV replication by inhibiting both IN-LEDGF/p75 interaction and IN dimerization. Analogs of 3-hydroxypicolinamides exhibited micromolar EC_50_ values against HIV replication in a cell-based assay [114].

#### CX14442

A quinoline derivative, CX14442 (**22**) is the first LEDGIN that was found to have antiretroviral potency in the low nanomolar range (EC_50_ value of 69 nM) and is superior to most quinoline derivatives reported so far [115]. It is a derivative of the BI-1001, a LEDGIN (**19**) [105]. The hydrophobic domains of BI-1001 (**19**) and CX14442 engages one subunit of HIV-1 IN CCD dimer through hydrophobic interactions and the hydrophilic groups form hydrogen bonds with the residues. Compared with BI-1001 (**19**), CX14442 has a larger tert-butyl group in lieu of the methyl group of the ester functionality. This modification demonstrated better interactions with the highly hydrophobic-binding pocket of CCD dimer interface and resulted in stronger affinity [105]. It inhibited ST step, but the catalytic activity of IN was not completely blocked [95]. The mutations Y99H, A128T and A129T were identified with CX14442 in resistance selection experiments [116].

#### N-aryl-naphthylamines

Screening an in-house *N*-aryl-naphthylamines chemical library, Giuliana *et al*. [117] identified several compounds with general structure **23** inhibiting LEDGF/p75 and IN interaction. Their activities (IC_50_) against IN-LEDGF interaction ranged from 70.07 and 2.5 μM. Compound **24**, 4-(4-hydroxy-1-naphthylamino)benzoic acid, was found to have the highest LEDGF/p75-IN inhibition with an IC_50_ value of 2.5 μM [117].

#### BI 224436

BI 224436 (**25**) is a 3-quinolineacetic acid derivative that was the first LEDGIN to advance into Phase IA clinical trials because of its good bioavailability, tolerability, pharmacokinetics and plasma levels [96,118]. It was discovered through a high-throughput 3′-P screening assay against the Boehringer Ingelheim compound library and is currently licensed to Gilead sciences for further development [83,119]. As an allosteric inhibitor, BI 224436 targets a noncatalytic site and disrupts chromatin and IN from interacting with LEDGF/p75 [83]. It was found to inhibit 3′-P step, IN interaction with LTR DNA and LEDGF without inhibiting the ST step. Crystallography studies have also found that BI 224436 binds to the highly conserved allosteric pocket of the CCD of IN, which is also the site where LEDGF binds [96].

BI 224436 (**25**) is currently in early clinical trials and is predicted to have low cross-resistance with other INIs because it binds to a distinct site on IN and was shown to retain effectiveness against high-level RAL-resistant strains. It was also the first INI identified to bind at a noncatalytic site [118]. Using an assay where the virus was passed *in vitro* in the presence of BI 224436, primary mutations A128T, A128N or L120F were selected. These residues contour the conserved, allosteric pocket within the CCD, confirming the fact that BI 224436 functions as an ALLINI. Interestingly, BI 224436 was found to retain its full antiviral activity against common INSTI mutations including N155S, Q148H and E92Q [96].

#### LEDGIN1 & LEDGIN6

These are quinoline-based compounds that inhibit LEDGF/p75-IN binding, IN catalytic activity and HIV-1 replication in cell culture. They were discovered through two different approaches. *In silico* pharmacophore screening of a commercial library of 200,000 compounds led to the discovery of LEDGIN1 (**26**). Another structurally similar compound BI-A (**28**), which is also an analog of BI-1001 (**19**) was identified about the same time by Fenwick and colleagues, through a high-throughput screening searching for inhibitors of IN 3′-P activity. However, neither compound was found to inhibit HIV-1 replication. They had promising *in vitro* activities: LEDGIN1 (**26**) at 100 μM inhibited LEDGF/p75-IN interaction by 36%, whereas at 9 μM BI-A (**28**) inhibited 50% of IN 3′-P activity. Moving forward with these lead compounds, inhibitors of HIV replication with EC_50_ values ranging from 10 to 100 nM were developed [120].

Analogs of LEDGIN1 (**26**) such as LEDGIN6 (CX0516) (**27**) was found to selectively impair IN-LEDGF/p75 binding. Using an array of assays, similar concentrations of BI-1001 (**19**) and LEDGIN6 (**27**) (e.g., 4–10 μM for **27**; 1–2 μM for **19**) inhibited both LEDGF/p75-IN binding and LEDGF/p75-independent IN 3′-P and DNA ST activities *in vitro* [120].

These inhibitors were cocrystalized at the LEDGF/p75-binding site, distant from the active site, and are thus known as ALLINIs. Key pharmacophore carboxylic acid mimics the bidentate H-bonding pattern of LEDGF/p75 residue Asp 366 with the IN polypeptide backbone. The presence of a *tert*-butoxy moiety moreover correlates with ALLINI potency [120].

LEDGIN6 (**27**) in particular was highly selective for disrupting IN-LEDGF binding (IC_50_ = 1.37 μM). LEDGIN-6 (**27**) and BI-1001 (**19**) are frequently grouped together because they have identical antiviral mechanisms and belong to the same class of inhibitors, which inhibit IN activities through compound-mediated premature protein multimerization. It is noted that BI-1001 (**19**) was more potent than LEDGIN6 (**27**) [111]. Although LEDGIN-6 did not inhibit 3′-P activity *in vitro*, Boehringer Ingelheim identified a remarkably similar series of compounds using a high-throughput screening for HIV-1 IN 3′-P activity. Both compounds similarly inhibited 3′-P and DNA ST activities in the absence of added LEDGF/p75 protein [121].

The resistance strain with A128T mutation was identified in a serial passaging experiments with LEDGIN 6 (**27**) as the inhibitor [122]. A128T is localized in the LEDGF-binding pocket [111]. Consequently, it was identified as a hot spot for IN-LEDGF/p75 interface and a primary resistance mutation to various LEDGINs [100]. Another potent LEDGIN that was identified with similar structure features was LEDGIN7 (CX05045) (**29**) [103].

#### MUT-A

**Figure 4.**
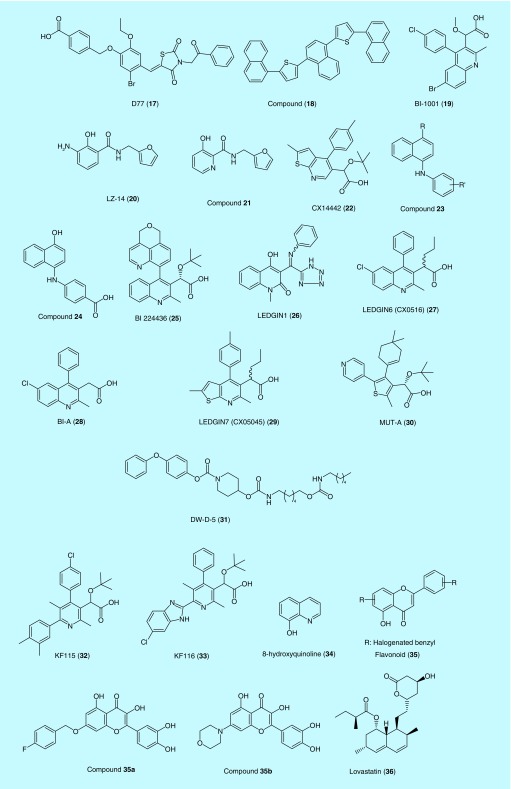
**Representative structures of allosteric integrase inhibitors.**

MUT-A (**30**) Figure 4 was discovered as a new type LEDGINs while optimizing a series of small molecules. It has structural similarities with other LEDGINs with a common pharmacophoric features including a *tert*-butylether motif linked at the α-position of a carboxylic acid and a thiophene ring. It has displayed a more potent antiretroviral activity compared with previously reported LEDGINs, but contains a similar profile of resistance mutations [123].

#### DW-D-5

Docking of compounds from the Maybridge database against the p75-binding site of HIV-1 IN led to identification of a series of compounds, in which DW-D-5 (**31**) showed promising antiviral properties with EC_50_ value of 2.0 μM in the inhibition of HIV-1 replication [124]. DW-D-5 inhibited the HIV-1 IN ST activity with an IC_50_ value of 0.85 μM. When combined with the FDA-approved HIV drugs, DW-D-5 resulted in additive inhibitory effect on HIV-1 replication [95,124]. For example, cotreatment of 1 μM DW-D-5 with 5 nM RAL exhibited 51.69% inhibitory effect, whereas RAL alone exhibited 35.34% inhibitory effect at the same dose. Optimization of DW-D-5 could offer a novel scaffold for LEDGINs development.

#### 1,4-Bis(5-(naphthalene-1-yl)thiophen-2-yl)naphthalene

1,4-Bis(5-(naphthalene-1-yl)thiophen-2-yl)naphthalene (**18**) was developed by Gu *et al*. as a small-molecule inhibitor of LEDGF/p75-IN interaction. Molecular docking simulations showed that compound **18** binds to the CCD hydrophobic pocket of IN in the proximity of the LEDGF-binding site. All eight rings, especially the six phenyl rings, showed strong hydrophobic interactions at the hydrophobic pocket. Binding disrupts IN-LEDGF interaction and inhibits IN nuclear translocation [99,125]. It suppressed viral replication with an EC_50_ value of 11.19 μM [126].

#### Multimeric INIs

In pursuit of INIs with multimodes of actions, much attention has focused on compounds that promote IN multimerization. The multimerization-selective HIV-1 INIs (MINIs) are a distinct class of allosteric INIs. MINIs potently inhibit HIV-1 replication during virion maturation by inducing hyper- or aberrant IN multimerization while they are largely ineffective during the early steps of viral replication. By altering the quinoline moiety in LEDGINs, new pyridine-based molecules were developed as MINIs. The MINIs have a rotatable single bond that bridges between interacting IN subunits and promotes oligomerization. Using available x-ray crystal structure of quinolone-based ALLINIs bound to IN CCD dimer, the existing scaffold was modified to enhance potency specificity for modulating multimerization without affecting IN-LEDGF/p75 binding. Using BI-1001 (**19**) (IC_50_ = 5.8 μM) as a structural prototype, two new compounds KF115 (**32**) and KF116 (**33**) were developed by altering the quinolone moiety into biaryl moiety that contains a pyridine ring. KF116 (**33**) with an EC_50_ value of 0.024 μM is being further investigated [110,127].

#### 8-Hydroxyquinolines

Quinolone derivatives are commonly used as antifungal, antibacterial and antitubercular drugs [128]. Molecular modeling studies revealed 8-hydroxyquinoline (**34**) bound to IN at the IN-LEDGF/p75 interface, inhibiting the required protein–protein interaction. Additional structural modifications improved its potency and produced IN-LEDGF/p75 interaction inhibitors. Their potency was sevenfold greater than that of compound **34**, with IC_50_ values ranging from 0.4 to 4.8 μM for IN-LEDGF/p75 interaction [129].

#### Flavonoid-based ALLINIs

Flavonoids are widely used class of natural products with variety of activities including anti-inflammatory, antioxidative and antiviral activities. β-ketoenol or catechol structure in some flavonoids are capable of chelating metals, a key structural requirement for INIs, resulted in the identification of a novel pharmacophore with a general structure as shown in compound **35.** Based on pharmacophore-based drug design and structure–activity relationship studies, a new series of 3/5/7/3′/4′-substituted flavonoid derivatives were developed. The derivatives were evaluated on inhibitory activity against enzyme, IN-LEDGF/p75 interaction and viral infection of C8166 cells. They had excellent inhibitory activities in enzyme-based assays and protected cells from infection in some cell-based assays. Hydroxyflavones blocked IN-LEDGF/p75 interaction at a low to submicromolar IC_50_ values and served as a novel scaffold in designing new drugs that target the catalytic site and LEDGF/p75 interaction. A flavonoid analog 2-(3,4-dihydroxyphenyl)-7-((4-fluorobenzyl)oxy)-3,5-dihydroxy-4*H*-chromen-4-one (**35a**) has the highest inhibitory activity with IC_50_ value of 4 μM against ST activity within this class. Substituting with a hydrophilic morpholino group at C7 position improved activity against LEDGF/p75-IN interaction with IC_50_ values ranging from 0.97 to 1.7 μM as seen in 2-(3,4-dihydroxyphenyl)-3,5-dihydroxy-7-morpholino-4*H*-chromen-4-one (**35b**) [130].

#### Lovastatin

Virtual screening and biological evaluation of antidyslipidemia statins identified some statins as potential LEDGF/p75-IN interaction inhibitors. Among the known eight stains, lovastatin (**36**) was the most potent statin with an IC_50_ of 1.97 μM against the protein–protein interaction. Unfortunately, none of the statins yielded antiviral activity *in vitro* [131].

### Dual inhibitors

In order to address resistance problems associated with the current HIV drug regimen, many new strategies have been developed. These include drugs that have sustained-release, polypills and dual inhibitors. The dual inhibitors contain a pharmacophore that allows it to bind to two different targets to produce additive or synergistic effects [132]. The following two different approaches were explored to target IN and RT enzymes, and IN catalytic site and LEDGF-binding site to develop dual inhibitors.

### IN & RT RNase H domain inhibitors

The most common type of dual inhibitors are those that target IN and RNase H of RT, which share a common catalytic site residues and geometry [75,133]. Both sites have a common αβ-fold that contains a central five-stranded mixed β-sheet next to α-helices on each side. In addition, the two enzymes have key acid amino acids (D64, D116 and E152 for HIV-1 IN; D443, E478, D498 and D549 for the RNase H domain) that have the ability to chelate two magnesium metal ions in their active sites [102]. As a result, compounds like DKA inhibitors and DNA aptamers that target RNase H also exhibit inhibitory activity against IN [134]. By targeting two major enzymes essential for HIV replication, it is promising that this novel strategy could offer better treatment regimens superior to early generations of anti HIV-1 regimens that generally required the combination of multiple antiviral drugs. Therefore, fewer side effects, less pill burden and increased drug adherence may be achieved.

#### 2-Hydroxyisoquinoline-1,3(2H,4H)-diones

A class of 2-hydroxyisoquinoline-1,3(2*H*, 4*H*)-diones (HIDs) with a general structure **37** were developed as novel IN and RT RNase H domain dual inhibitors. HIDs were found to have high genetic barrier and favorable cross-resistance profiles with RAL. HIDs work by inhibiting both ST and 3′-P activities. The scaffold contains three Mg^2+^-chelating oxygen atoms that form a monocyclic ring, making it difficult to select replicating resistant variants [135]. Several analogs of **37** were synthesized and found with IC_50_ values ranging from 10 to 260 nM [135,136]. All compounds within this class exhibited inhibitory activities against RT RNase H and IN but displayed high cytotoxicity in cell culture [137]. Thus, it was deemed that further optimization of the scaffold is necessary before this class of compounds can be translated into clinical candidates.

#### Quinolinonyl DKA derivatives

A series of basic quinolinonyl DKA derivatives were developed as dual inhibitors of IN and RNase [138]. Their ST activities were in low micromolar to nanomolar range. Among the reported compounds, **38** (4-[1-[(4-fluorophenyl)methyl]-7-[*N*-(3-chloroprop-1-yl)piperazin1-yl]-4-(1H)-quinolinon-3-yl]-2-hydroxy-4-oxo-2-butenoic acid) inhibited the ST step with an IC_50_ value below 50 nM and showed a two order of magnitude selectivity over 3′-P. The compound **38** also displayed RNase H activity with IC_50_ value of 5.7 μM.

Later, *N*-substituted quinolinonyl DKA derivatives were reported as IN and RT RNase H in inhibitors [139]. These analogs provided an opportunity to investigate the role of the arylmethyl group linked to quinolonyl nitrogen. Several of these analogs were found to be potent dual INIs showing high activity against the ST and RNase H function. Compound **39** (4-[1-(2,4-difluorophenyl)methyl-4(1*H*)-quinolinon-3-yl]-2-hydroxy-4-oxo-2-butenoic acid) displayed the highest ST activity with IC_50_ value of 10 nM and RNase H activity with an IC_50_ value of approximately 36 μM [139].

#### Pyrrolyl derivatives

In search of dual inhibitors, Costi *et al*. reported a series of 6-(1-benzyl-1H-pyrrol-2-yl)-2,4-dioxo-5-hexenoic acid derivatives as IN and RNase H inhibitors [140]. Over 50 analogs were synthesized and tested on recombinant enzymes (IN and RNase H) in cell-based assays. Approximately 22 compounds exhibited inhibition of HIV replication. Three compounds were active against RNase H activity with IC_50_ values ranging from 2.5 to 3.0 μM. The compound **40** was found to be the most potent INI with IC_50_ value of 26 nM. In the meantime, compound **40** displayed RNase H activity with IC_50_ value of 2.5 μM [140].

Pyrrolyl DKA derivatives were further explored to optimize dual inhibitors against IN and RNase H [141]. Two classes of pyrrolyl DKA derivatives were reported: the diketobutanoic and the diketohexenoic derivatives. Both classes of compounds exhibited good potency against IN and a moderate inhibition against RNase H. Among all the diketohexenoic derivative **41** (ethyl 6-(4-(4-fluorobenzoyl)-1-phenyl-1*H*-pyrrol-2-yl)-2-hydroxy-4-oxohexa-2,5-dienoate) was the most active compound against IN and RNase H with IC_50_ values of 1.8 and 1.2 μM, respectively.

### IN & LEDGF/p75-IN interaction inhibitors

#### CHI-1043

Based on the DKA pharmacophore, three indole derivatives and their magnesium(II) complexes were studied for their ability to inhibit both integration and IN-LEDGF/p75 interaction. In particular, CHI-1043 (**42**) was found to have decent activity in both enzymatic and cellular assays with low toxicity. It had an IC_50_ value of 0.04 μM in enzymatic and cell-based assays. Docking studies showed that CHI-1043 and its analogs bind similarly to INSTIs with the DKA moiety coordinating the two metal cofactors and forming metal complexes with different stoichiometric ratios. The metal complexes inhibited IN at low nanomolar to micromolar concentrations and both the complexes and free ligands inhibited IN-LEDGF/p75 interaction at low micromolar values. Because of the good antiviral activities observed by these magnesium compounds, metal coordination was suggested as a new design for antivirals [142].

#### CHIBA-3002, CHIBA-3003 & CHIBA-3053

Compounds CHIBA-3002***(43)***, CHIBA-3003***(44)*** and CHIBA-3053***(45)*** were found to inhibit HIV-1 IN LEDGF/p75 interaction at micromolar concentrations [143]. CHI-1043 inhibited both the catalytic site of IN to affect ST reaction and IN-LEDGF/p75 interaction. A series of new dual inhibitors created with a general structure (**46**) by combining the scaffold of CHIBA and CHI analogs, specifically by adding the indole β-DKA scaffold present for IN-LEDGF interaction inhibitors and important pharmacophoric characteristics of INSTIs [144].

#### CX04328

**Figure 5.**
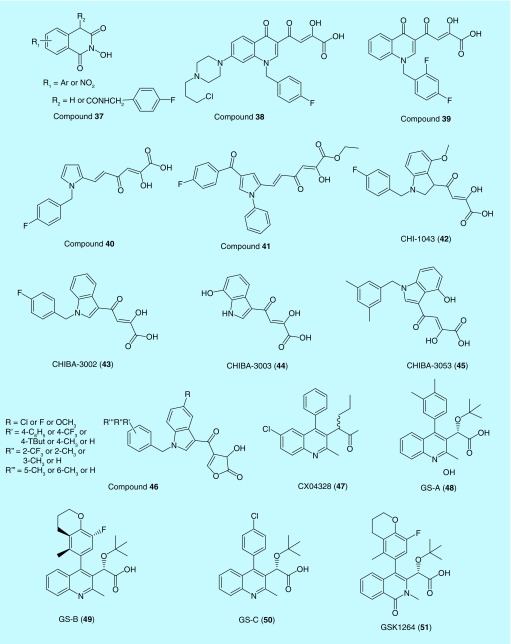
**Representative structures of dual HIV inhibitors.**

Pharmacophore hypothesis and virtual screening of a database of 200,000 compounds resulted in the identification of a series of dual inhibitors. The most potent compound against IN-LEDGF/p75 interaction was found to be (2S)-2-(6-chloro-2-methyl-4-phenylquinolin-3-yl)pentanoic acid (CX04328) (**47**) Figure 5. CX04328 binds to a cleft between two monomers of the IN core dimmer [106]. AlphaScreen assays showed an IC_50_ value of 1.37 μM for IN inhibition. It also had EC_50_ values of 2.73 and 3.45 μM when tested against infected MT-4 or PBMCs, respectively (Peat). CX04328 retained full activity against RAL-resistant mutants. However, cell lines overexpressing LEDGF/p75_IBD_ with A128T/Q170G double mutations conferred resistance [106].

#### Tert-butoxy(4-phenyl-quinolin-3-yl)-acetic acid

Another class of compounds developed based on quinoline pharmacophore were *tert*-butoxy(4-phenyl-quinolin-3-yl)-acetic acids. *Tert*-butoxy(4-phenyl-quinolin-3-yl)-acetic acids work as a competitive inhibitors against LEDGF and disrupt the proper tethering of the IN on the chromatin. In addition, they cause conformational changes in the IN dimer and inhibit the donor DNA from forming a proper complex with IN.

Three compounds within this series, coded GS-A (**48**), GS-B (**49**) and GS-C (**50**) showed potent antiviral activities with EC_50_ values ranging from 10 to 287 nM. Crystal structures of GS-A (**48**) and GS-B (**49**) showed their binding at IN CCD dimer interface inhibiting IN-LEDGF interaction making them true LEDGINs. Their IC_50_ values were ranging from 19 to 228 nM. They also promoted IN dimerization, causing the enzyme to lose flexibility and pushing it to an inactive state. Hence, these compounds have a multimodal mechanism, inhibiting LEDGF/IN interaction as well as promoting IN dimerization leading to an inactive enzyme [145]. These constitute a new class of inhibitors that are structurally distinct from IN ST inhibitors but analogous to LEDGINs [109,145].

#### GSK1264

GSK1264 (**51**) is also a dual inhibitor inhibiting LEDGF/IN interaction as well as 3′-P by promoting IN polymerization. It forms insoluble LEDGF/IN aggregates. Cocrystal structure of GSK1264 (**51**) revealed that it binds to α1 and α3 helices of the first monomer subunit, and α4 and α5 helices of the second monomer subunit of IN. Interestingly, this compound inhibits late stages of viral replication postintegration, rather than early. Such inhibition still allowed the virus to be released from the host cell; however, the new virions were disabled from infecting new host cells [146].

## Conclusion

This review summarizes recent advances up to 2018 in the field of HIV-1 IN inhibitors in connection to major compounds that have made it into clinical trials. It does not address all literature published in the year but attempts to include major advances. The information presented serves to give readers a sense of where research in the field is heading at the current point in time.

## Future perspective

Although the research for HIV-1 INIs is relatively new and has progressed at a fast pace, there has been a significant reduction in the number of compounds advancing into clinical trials. In order to tackle the problems associated with resistance, many research groups have made efforts to discover novel targets or mechanism of action. To date, the dual inhibitors targeting the HIV-1 IN and RT emerged as the most promising class of HIV-1 INIs because their capacity to interact with two targets makes it difficult for the virus to develop resistance. However, the virus has a very high turnover rate – billions of copies every day. Small genetic errors or mutations can lead to easy development of drug resistance. Thus, the pursuit of new anti HIV-1 agents is always in competition with the development of drug resistance.

**Executive summary**

**HIV integrase**

- A key viral enzyme in HIV replication facilitates integration of the viral genome with the host genome.
- This makes it an attractive target for drug development, as it does not have any host cellular homolog.
- It functions by interacting with other viral as well as host cellular cofactors including lens EDGF (LEDGF/p75), p300 histone acetyl transferase (HAT), etc.

**HIV integrase inhibitors**

- β-Diketo acid derivatives were the first promising class of HIV integrase (IN) inhibitors.
- Divalent metal chelating functional groups are required for strand transfer inhibitors to chelate two magnesium metals present in the active site of catalytic core domain.
- First-generation IN inhibitors such as raltegravir, elvitegravir and the second-generation inhibitor dolutegravir evolved from β-diketo acids scaffold. All of the developed inhibitors predominantly inhibit the strand transfer step.
- Several other strand transfer inhibitors such as GS-9160, MK-2048, cabotegravir and bictegravir followed to address the resistance problems encountered by the first three drugs.
- Allosteric IN inhibitors (ALLINs) were developed to address resistance and cross-resistance problems encountered by IN strand transfer inhibitors, especially by inhibiting its interaction with one the essential cofactors, LEDGF.
- Several LEDGF/p75-IN interaction inhibitors (LEDGINs) at various stages of development were reported.
- Dual inhibitors such as IN and reverse transcriptase RNase H domain inhibitors and IN and LEDGF/p75-IN Interaction inhibitors are explored to tackle the resistance problems from the earlier candidates.
- Several promising dual inhibitors are reported in this review.
